# Estimation of free water-corrected microscopic fractional anisotropy

**DOI:** 10.3389/fnins.2023.1074730

**Published:** 2023-03-07

**Authors:** Nico J. J. Arezza, Tales Santini, Mohammad Omer, Corey A. Baron

**Affiliations:** ^1^Department of Medical Biophysics, Schulich School of Medicine and Dentistry, Western University, London, ON, Canada; ^2^Centre for Functional and Metabolic Mapping (CFMM), Robarts Research Institute, Western University, London, ON, Canada

**Keywords:** diffusion, MRI, microscopic anisotropy, free water elimination, microstructure, neuroimaging, cerebrospinal fluid

## Abstract

Water diffusion anisotropy MRI is sensitive to microstructural changes in the brain that are hallmarks of various neurological conditions. However, conventional metrics like fractional anisotropy are confounded by neuron fiber orientation dispersion, and the relatively low resolution of diffusion-weighted MRI gives rise to significant free water partial volume effects in many brain regions that are adjacent to cerebrospinal fluid. Microscopic fractional anisotropy is a recent metric that can report water diffusion anisotropy independent of neuron fiber orientation dispersion but is still susceptible to free water contamination. In this paper, we present a free water elimination (FWE) technique to estimate microscopic fractional anisotropy and other related diffusion indices by implementing a signal representation in which the MRI signal within a voxel is assumed to come from two distinct sources: a tissue compartment and a free water compartment. A two-part algorithm is proposed to rapidly fit a set of diffusion-weighted MRI volumes containing both linear- and spherical-tensor encoding acquisitions to the representation. Simulations and *in vivo* acquisitions with four healthy volunteers indicated that the FWE method may be a feasible technique for measuring microscopic fractional anisotropy and other indices with greater specificity to neural tissue characteristics than conventional methods.

## Introduction

Diffusion-weighted MRI (dMRI) is a non-invasive imaging modality that uses specialized pulse sequences to sensitize the MRI signal to the random molecular motion of water (Stejskal and Tanner, 1965; Tanner, 1965). On MRI-relevant time frames, water molecules traverse microscopic length scales in tissue, and their diffusion is dictated by the presence of restricting boundaries such as cell membranes and other structures. By exploiting the known relationships between dMRI signal and tissue properties, dMRI measurements can act as surrogate indicators of physical properties of neural tissue, and this capability has led to dMRI finding use in the study of neurological disorders like multiple sclerosis (Rovaris et al., 2005; Inglese and Bester, 2010), Alzheimer’s disease (Zhang et al., 2009), and stroke (van Everdingen et al., 1998), among others.

The most widely used dMRI technique is diffusion tensor imaging (DTI). DTI is based on the first order cumulant expansion of the logarithm of the dMRI signal as a function of diffusion weighting or b-value (Basser et al., 1994; Frisken, 2001), which can be represented by the equation:


(1) $$S_{g,b}=S_{0}\,e^{-b\,\sum_{i,j=1}^{3}g_{i}\,g_{j}\,D_{i\,j}}$$

where *S*_**g**,*b*_ is the diffusion-weighted MRI signal of a particular acquisition acquired with diffusion-weighting applied in the direction of the unit vector ***g*** = (*g*_1_, *g*_2_, *g*_3_), *S_0_* is the MRI signal in the absence of diffusion weighting, *b* is the *b*-value, which describes the strength of the diffusion weighting, and *D*_*ij*_ is *ij^th^* element of the fully symmetric second order diffusion tensor, **D**. DTI requires linear tensor encoding (LTE) acquisitions in different diffusion directions at a single *b*-value plus one or more acquisitions with no diffusion weighting and can report metrics such as the mean diffusivity (MD) and fractional anisotropy (FA) of water diffusion. However, the DTI representation assumes that diffusion follows a mono-Gaussian distribution, which is a reasonable assumption only at low b-values (Johansen-Berg and Behrens, 2009). The diffusion kurtosis imaging (DKI) representation further expands the cumulant expansion of the logarithm of the dMRI signal to the second order to account for non-Gaussian diffusion but requires the acquisition of dMRI data at two or more *b*-values. The DKI model can be represented as (Jensen et al., 2005; Lu et al., 2006):


(2) $$S_{g,b}=S_{0}\,e^{-b\,\sum_{i,j=1}^{3}g_{i}\,g_{j}\,D_{i\,j}+\frac{1}{6}\,b^{2}\,\sum_{i,j,k,l=1}^{3}g_{i}\,g_{j}\,g_{k}\,g_{l}\,W_{i\,j\,k\,l}+O\,\left(b^{3}\right)}$$

where *W*_*ijkl*_ denotes the *ijkl^th^* element of the fully symmetric fourth order diffusion kurtosis tensor, **W**, and *O*(*b*^3^) is a higher order term that is negligible in brain tissue at b-values lower than 3,000 s/mm^2^ (Jensen and Helpern, 2010). The powder kurtosis signal representation (paK), in which data acquired from many diffusion directions are arithmetically averaged into a single image volume known as the powder average, can be represented as:


(3) $$S_{b}=S_{0}\,e^{-b\,D_{e\,f\,f}+\frac{1}{6}\,b^{2}\,D_{e\,f\,f}^{2}\,K+O\,\left(b^{3}\right)}$$

where *S_b_* is the dMRI signal of the powder averaged data at a particular b-value, *D*_*eff*_ is the effective diffusivity estimated from the powder average signals, and *K* is the effective diffusion kurtosis (Jensen et al., 2005; Lu et al., 2006). Note that diffusion metrics acquired from the powder representation (e.g., *D*_*eff*_) differ from similarly-named metrics acquired from the tensor representation (e.g., MD) (Henriques et al., 2021).

The DTI and DKI representations are limited by two major factors that affect their specificity to neuronal microstructure: (1) the tensors used to estimate anisotropy are sensitive to neuron fiber orientation dispersion within the voxel, causing FA to be reduced in brain regions containing crossing or fanning axons (Jones et al., 2013; Szczepankiewicz et al., 2015), and (2) the presence of cerebrospinal fluid and other free water pools (e.g., cysts) biases diffusion measurements in both the tensor and powder representations, potentially confounding or masking true microstructural changes within the tissue (Alexander et al., 2001; Jones and Cercignani, 2010; Vos et al., 2011; Baron and Beaulieu, 2015). Typically, a voxel with these free water partial volume effects will have elevated MD and reduced FA due to the high diffusivity and negligible anisotropy of free water.

To overcome the first limitation, techniques such as microscopic fractional anisotropy (μFA) imaging, which reports water diffusion anisotropy independent of the neuron fiber orientation dispersion, were developed (Jespersen et al., 2013; Lasič et al., 2014; Shemesh et al., 2016). μFA can be estimated by fitting traditional LTE dMRI data to various signal representations using *a priori* knowledge of the underlying tissue (Kaden et al., 2016a,b; Novikov et al., 2019) or by using advanced dMRI pulse sequences like double diffusion encoding (Cory et al., 1990; Henriques et al., 2020) or spherical tensor encoding (STE) (Lasič et al., 2014; Szczepankiewicz et al., 2015; Westin et al., 2016). Previous studies have demonstrated that μFA may be more suitable than FA for a number of applications such as in evaluating white matter degeneration in Parkinson’s disease (Ikenouchi et al., 2020), delineating lesions and detecting abnormalities in multiple sclerosis (Yang et al., 2018; Andersen et al., 2020), and differentiating between different types of brain tumors (Szczepankiewicz et al., 2015).

**FIGURE 1:**
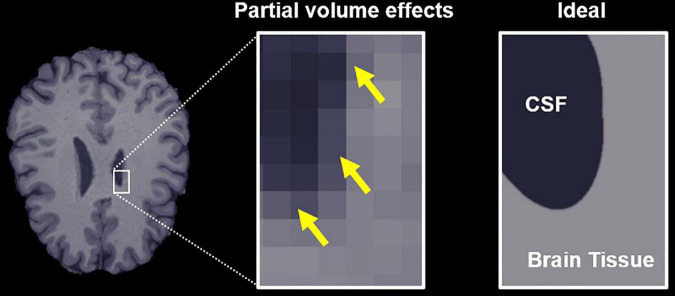
Free water partial volume effects at the interface between brain tissue and a ventricle containing cerebrospinal fluid (CSF). The image on the right depicts an ideal slice in which the brain tissue and CSF are clearly delineated, while the center image depicts partial volume effects in voxels that contain both CSF and tissue, highlighted by yellow arrows. The goal of the proposed algorithm is to obtain parameter estimates specific to the tissue in these voxels.

The bias caused by free water partial volume effects on DTI and DKI measurements results from the fact that indices quantified using both representations represent the weighted average of all water diffusion within a voxel rather than markers of a specific tissue. The diffusivity of free or unhindered water at 37°C is isotropic and approximately 3–4 times higher than that of brain tissue, so it has a significant effect on the voxel-level dMRI parameters, even at low volume fractions (Pierpaoli and Basser, 1996). Moreover, the free water signal is typically a factor of 2–3 times higher than brain-tissue for the T_2_-weighted scans used for dMRI, which further exacerbates these partial volume effects. Accordingly, dMRI measurements made in brain regions with significant free water partial volumes (Figure 1), such as the fornix and other ventricle-adjacent regions, are greatly affected (Metzler-Baddeley et al., 2012; Li et al., 2013).

The effects of free water partial volumes can be attenuated by using non-zero minimum diffusion weighting (Baron and Beaulieu, 2015) and by implementing fluid-attenuated inversion recovery dMRI sequences (Papadakis et al., 2002; Chou et al., 2005), but both techniques decrease signal-to-noise ratio (SNR), the former affects DTI metrics in tissue with minimal free water, and the latter increases specific absorption rate and scan time (Pasternak et al., 2009). Alternatively, modifications to the DTI and DKI representations can be used to distinguish between dMRI signal from free water and dMRI signal from functional brain tissue. The free water elimination DTI (FWE-DTI) representation separates the dMRI signal into two macroscopic components: one representing free water and one representing brain tissue (Pasternak et al., 2009), and can be expressed as:


(4) $$S_{b}=S_{0}\,\left(f\,e^{-b\,\sum_{i,j=1}^{3}g_{i}\,g_{j}\,D_{T,i\,j}}+\left(1-f\right)\,e^{-b\,\left(3\,e-3\right)}\right)$$

where *f* is the apparent volume fraction of tissue (weighted by differences in S_0_ between free water and tissue) within the voxel of interest and *D*_*T,ij*_ is the *ij^th^* element of the diffusion tensor corresponding to the tissue component (**D_T_**). The *(3e-3)* term represents the diffusivity of free water at 37°C in mm^2^/s. Note that signal arising from extracellular water that is hindered, such as the water between neuronal axons, will primarily contribute to the tissue component and not the free water component. This representation enables more accurate estimation of tissue-specific indices than traditional DTI and has attracted interest for use in studying neurodegeneration in Alzheimer’s disease (Hoy et al., 2017), Parkinson’s disease (Planetta et al., 2016), and traumatic brain injury (Pasternak et al., 2014a), among others. Additionally, the volume fraction metric is a potential surrogate marker for edema (Pasternak et al., 2009, 2014b). While traditional DTI can be performed using single b-shell data, FWE-DTI should be performed with data collected at multiple b-values to reduce model fitting degeneracies at the expense of increased scan time (Golub et al., 2020). Recently, the FWE-DTI representation was expanded to account for non-Gaussian diffusion in the tissue compartment by expanding the cumulant expansion to the second order; this modification to FWE-DTI is referred to as the free water elimination DKI representation (Collier et al., 2018).

In this article, we propose a technique to measure water diffusion anisotropy that combines the STE-based μFA acquisition protocol to achieve insensitivity to neurite orientation (Lasič et al., 2014; Szczepankiewicz et al., 2015) with the free water elimination representations’ ability to distinguish between free water partial volume effects and true tissue properties.

## Materials and methods

Previously, we demonstrated that μFA can be estimated by jointly fitting multi-shell LTE and STE dMRI data to the powder average diffusion kurtosis representation, as per the following equations (Arezza et al., 2021):


(5) $$S_{b,L\,T\,E}=S_{0}\,e^{-b\,D_{e\,f\,f}+\frac{b^{2}\,D_{e\,f\,f}^{2}\,K_{L\,T\,E}}{6}}$$

(6) $$S_{b,S\,T\,E}=S_{0}\,e^{-b\,D_{e\,f\,f}+\frac{b^{2}\,D_{e\,f\,f}^{2}\,K_{S\,T\,E}}{6}}$$

(7) $$\mu \,F\,A=\sqrt{\frac{3}{2}}\,{\left(1+\frac{6}{5\,\left(K_{L\,T\,E}-K_{S\,T\,E}\right)}\right)}^{-\frac{1}{2}}$$

where the subscripts *LTE* and *STE* denote the encoding scheme. By combining equations (5) and (6) with a FWE representation, the powder average free water elimination kurtosis representation (FWE-paK) can be defined in the LTE and STE encoding schemes *via* the following equations:


(8) $$S_{b,L\,T\,E}=S_{0}\,\left(f\,e^{-b\,D_{T}+\frac{b^{2}\,D_{T}^{2}\,K_{L\,T\,E}}{6}}+\left(1-f\right)\,e^{-b\,\left(3\,e-3\right)}\right)$$

(9) $$S_{b,S\,T\,E}=S_{0}\,\left(f\,e^{-b\,D_{T}+\frac{b^{2}\,D_{T}^{2}\,K_{S\,T\,E}}{6}}+\left(1-f\right)\,e^{-b\,\left(3\,e-3\right)}\right)$$

where *D_T_* is the effective diffusivity in the tissue compartment, *K*_*LTE*_ is the effective diffusion kurtosis in the tissue compartment in the LTE scheme, and *K*_*STE*_ is the effective diffusion kurtosis in the tissue compartment in the STE scheme. The *D_T_*, *K*_*LTE*_, and *K*_*STE*_ terms obtained using equations (8) and (9) characterize water diffusion in brain tissue independent of free water. Accordingly, μFA estimated from equation (7) using these corrected indices should characterize water diffusion anisotropy in tissue free of the bias caused by free water partial volumes. This imaging strategy which combines the FWE-paK signal representation with μFA imaging acquisition will be referred to herein as the FWE imaging method, whereas the technique that involves fitting the data to the powder kurtosis representation will be referred to as the conventional method.

### Fitting algorithm

In this work, a two-part algorithm (denoted Part I and Part II) was used to obtain a solution to the joint fitting of STE and LTE data. In the first part of the algorithm, low b-value (*b* ≤ 1,000 s/mm^2^) powder average STE data were fitted to a FWE representation for effective powder average diffusivity (FWE-paD) to obtain estimates of *f* and *D_T_*. The equation was derived from equation (9) by setting *K*_*STE*_ = 0:


(10) $$S_{b,S\,T\,E}=S_{0}\,\left(f\,e^{-b\,D_{T}}+\left(1-f\right)\,e^{-b\,\left(3\,e-3\right)}\right)$$

The indices computed with equation (10) were used as initial guesses in the second part of the algorithm, in which powder average STE and powder average LTE data across all *b*-values were jointly fitted to equations (8) and (9).

Part I of the algorithm exploits the FWE-paD representation’s lower complexity relative to the FWE-paK representation, reducing the number of unknown variables to be solved for by omitting the effective kurtosis term. The effects of non-Gaussian diffusion on dMRI, while deleterious to signal representations based on the first order cumulant expansion of the dMRI signal, are minimal at low b-values; thus, *f* and *D_T_* can be initially estimated despite omitting the second order term in the cumulant expansion. Using only the STE data as input further reduces the effects of non-Gaussian diffusion on the fit because it typically has minimal kurtosis. More specifically, LTE introduces a variance to the powder average signal due to the different diffusion encoding directions used for each acquisition; STE signals are free of this variance and deviate less from the mono-Gaussian diffusion assumption inherent to the FWE-paD signal representation in tissue-containing voxels (Lasič et al., 2014; Henriques et al., 2020). In this work, an iterative method was used to solve the FWE-paD equation. In each iteration, the low b-value STE data were first fitted to the FWE-paD representation [equation (10)] using the least squares method with a fixed estimate of *D*_*T*_ = 7*e*−4*mm*^2^/*s* used as an initial guess in the first iteration. Then, a correction was implemented to constrain *f* and (1−*f*) to be positive. The *D_T_* estimate was then updated by again fitting the data to equation (10) using the least squares method, this time with *f* and (1−*f*) as fixed inputs. A correction was implemented at the end of each iteration to set *D_T_* to 0 in voxels with very small tissue compartments (*f* < 0.1). The FWE-paD fit performed in Part I could be replaced by other techniques to obtain initial estimates of *f* and *D_T_* depending on data availability; for example, if low b-value STE data is not available, LTE data can be fitted to the FWE-DTI model depicted in equation (4).

In Part II of the algorithm, the LTE and STE data across all b-values were jointly fitted to the FWE-paK representation using the *f* and *D_T_* indices from Part I as initial estimates. Again, an iterative method was employed that was similar to that of Part I. In each iteration, the data were first fitted to the equations (8) and (9) to solve for *D_T_*, *K*_*LTE*_ and *K*_*STE*_ using a fixed *f* value (the first iteration used the value of *f* that was obtained from Part 1). Corrections were performed to constrain *K*_*LTE*_ to be positive and *K*_*STE*_ to be greater than or equal to −0.1. Then, the data were jointly fitted to equations (8) and (9), this time using fixed estimates of *D_T_*, *K*_*LTE*_ and *K*_*STE*_ to obtain an updated estimate of *f*. A final correction was performed at the end of each iteration to constrain *f* and (1−*f*) to be positive.

Part I and Part II were each performed for 100 iterations for all simulated and *in vivo* implementations of FWE-μFA investigated in this article. For all cases, adding more iterations caused negligible changes in the output parameters. The fitting code is openly available at gitlab.com/coreybaron/fwe_ufa.

### Synthetic dMRI simulations

To investigate the differences between the FWE-μFA method proposed herein and standard fitting, equations (8) and (9) were used to generate synthetic LTE and STE powder average signals to simulate white matter (WM) and gray matter (GM) voxels. These simulations were designed to also probe the performance of the non-convex fitting algorithm under the influence of noise and incorrect estimates for the free water diffusivity. For each voxel, signals were generated for b-values of 0, 700, 1,000, 1,400, and 2,000 s/mm^2^. To simulate a typical WM configuration, μFA was measured from publicly available dMRI data (Baron and Arezza, 2020) using the conventional μFA method (Arezza et al., 2021), and typical parameter values were extracted from frontal WM voxels in which free water contamination is expected to be minimal relative to tissue in other brain regions. The corresponding parameters are *D*_*T*_ = 8*e*−4*mm*^2^/*s*, *K*_*LTE*_ = 1.2, and *K*_*STE*_ = 0.1, which corresponds to a μFA of 0.85 as per equation (7). These parameters were used to simulate the signal acquired in voxels with simulated tissue volume fractions (*f*_*sim*_) of 0.2, 0.4, 0.6, 0.8, and 1 *via* equations (8) and (9). Rician noise was simulated by adding random Gaussian noise to the real and imaginary components of the signal and then computing the magnitude of the noisy signal. The standard deviation of the noise added to the signals was scaled by $1/\sqrt{N_{a\,c\,q}\,\left(b\right)}$, where *N*_*acq*_(*b*) is the number of acquisitions used experimentally (refer to Section “Materials and methods: *In vivo*”) for each b-value, to account for averaging from multiple acquisitions when the powder average is computed. Note that the noise standard deviation was chosen to achieve a specific SNR for the case in which *f*_*sim*_ = 1 and the b-value is 0. WM voxels were simulated at SNR values of 10, 20, and 40 (before scaling noise based on the number of acquisitions) with a fixed free water diffusivity of 3*e*^−3^ *mm*^2^/*s* to assess the effects of noise on the measurements. Also, WM voxels were simulated with free water diffusivities of 2.85*e*−3 and 3.15*e*^−3^ *mm*^2^/*s* at the SNR of 20 to assess how deviations in free water diffusivity affect the measurements. Notably, principal component analysis (PCA) denoising (Veraart et al., 2016) is typically used for *in vivo* data prior to parameter fitting and, accordingly, the simulations likely explore a more challenging fitting scenario than *in vivo*.

Due to the presence of free water in cortical GM voxels, as well as the heterogeneity between different deep GM regions of the brain, a typical GM configuration is difficult to assess. For this work, GM μFA was set to 0.55 as this is within the range of values found in the hippocampus (Yoo et al., 2021) and other deep GM regions (Lawrenz et al., 2016); using the same *D_T_* as the WM simulations, the *K*_*LTE*_ and *K*_*STE*_ values were set to 0.9 and 0.6, which yields the desired μFA = 0.55 *via* equation (7). GM simulations were performed over the same tissue volume fractions, SNRs, and free water diffusivities as the WM simulations.

A total of 1,000 realizations of random noise were generated for each simulation configuration. The FWE and conventional methods of estimating μFA were performed on the simulated voxels, and the mean and standard deviation of the following indices were computed across all setups for both methods: *D_T_*, effective anisotropic kurtosis (*K*_*aniso*_), effective isotropic kurtosis (*K*_*iso*_), and μFA. The effective kurtosis terms were computed as follows:


(11) $$K_{a\,n\,i\,s\,o}=K_{L\,T\,E}-K_{S\,T\,E}$$

(12) $$K_{i\,s\,o}=K_{S\,T\,E}$$

The relative error against the known ground truth was computed for each measurement using the following equation:


(13) $$R\,e\,l.E\,r\,r\,o\,r=\frac{X_{m\,e\,a\,s}-X_{G\,T}}{X_{G\,T}}$$

where *X* is the metric of interest and the subscripts *meas* and *GT* denote the measured value and known ground truth, respectively.

### Monte Carlo simulations

The synthetic powder average signals simulated in the previous section were derived using the same equation as is used in Part II of the fitting algorithm, which may glamorize the FWE technique. To validate those results, Monte Carlo random walk simulations were performed using Camino (Cook et al., 2006) to compare FWE with the conventional signal representation in a scenario in which the ground truth was known. The simulation geometry was set to be infinitely long cylinders to represent neuronal axons with a 1 μm radius, 0.7 intra-tube volume fraction, and water diffusivity of 2*e*−3*mm*^2^/*s* (Dhital et al., 2019); note that this case is assumed to represent a tissue volume fraction of *f*_*sim*_ = 1 as the extra-tube water is restricted and thought to contribute to *D_T_*. A free water compartment was simulated using a diffusivity of 3*e*−3*mm*^2^/*s* and was added to the tissue to achieve *f*_*sim*_ values of 0.2, 0.4, 0.6, 0.8, and 1. LTE and STE signals were simulated at b-values of 0, 700, 1,000, 1,400, and 2,000 s/mm^2^, with 15 diffusion directions acquired at each b-value in the LTE scheme.

LTE and STE data were powder averaged and the following metrics were estimated using the conventional and FWE methods: *D_T_*, *K*_*aniso*_, *K*_*iso*_, and μFA. The metrics computed using the conventional paK method at *f*_*sim*_ = 1 were assumed to be the ground truth and were used to compute the relative error for all other measurements.

### In vivo

To assess the FWE μFA algorithm in real dMRI data, four healthy volunteers (two female and two male, mean age 28.0 ± 6.6 years) were scanned on a 3T Prisma whole body MRI system (Siemens, Munich, Germany) located in the Center for Functional and Metabolic Mapping at Western University with 80 mT/m strength and 200 T/m/s slew rate. Volunteers first underwent T1-weighted MPRAGE acquisitions with 1 mm isotropic resolution to provide structural image volumes for segmenting regions-of-interest (ROIs). Then each subject underwent dMRI scans consisting of five acquisitions with no diffusion-weighting (*b* = 0 s/mm^2^), and 3, 15, 6, and 22 LTE acquisitions plus 6, 10, 10, and 27 STE acquisitions at b-values of 700, 1,000, 1,400, and 2,000 s/mm^2^, respectively. The STE pulse sequence used is described in Arezza et al. (2021). The other parameters for the dMRI acquisitions were: TE/TR = 94/4,500 ms, field-of-view = 220 × 200 mm^2^, resolution = 2 mm (isotropic), 48 slices, and rate 2 in-plane parallel imaging combined with rate 2 simultaneous multislice. Note that the b-values acquired in the dMRI acquisitions match those of the synthetic dMRI and Monte Carlo simulations.

Post-processing for the dMRI data included PCA denoising (Veraart et al., 2016) and Gibbs ringing correction using MRtrix3 (Kellner et al., 2016; Tournier et al., 2019), and eddy current artifact correction using FSL Eddy (Andersson and Sotiropoulos, 2016). Powder average signals were then computed from the LTE and STE data at each b-value and were then fitted to equations (8) and (9) to obtain μFA *via* the FWE method and fitted to equations (5) and (6) using ordinary least squares to obtain μFA *via* the conventional method.

The T1-weighted image volumes were used to obtain masks for ROIs because of their superior resolution and soft-tissue contrast compared to the dMRI image volumes. WM ROI masks were generated using the FAST tool from FSL (Zhang et al., 2001) using a probability threshold of 99% and limiting the masks to the region of the brain superior to the thalamus. Masks for the hippocampus, putamen, and thalamus were generated using the FIRST tool from FSL (Patenaude et al., 2011). ROI masks for the fornix were manually drawn. The T1 volumes were then registered to the powder averaged *b* = 0 s/mm^2^ volumes using symmetric diffeomorphic and affine transformations with ANTS software;^1^ these transformations were then applied to each of the ROI masks to register them to dMRI space.

The ROIs were selected to test several specific hypotheses. The WM and putamen are generally less contaminated by free cerebrospinal fluid than other regions, so it was expected that measurements made with the FWE and kurtosis μFA methods would be similar. The thalamus and hippocampus ROIs represent deep GM structures adjacent to free water, in which it was expected that the *D_T_* would be reduced and μFA would be elevated when using the FWE technique due to mitigation of free water signal. The fornix, which is both adjacent to the lateral ventricles and small relative to the image resolution, represents an ROI that is likely to have significant free water contamination; thus, much lower *D_T_* and much higher μFA were expected in this region when the FWE technique was used.

Mean and standard deviation of the following indices were computed in each of the ROIs to compare the FWE and conventional μFA techniques: *D_T_*, *K*_*aniso*_, *K*_*iso*_, and μFA. Voxels with *f* < 0.25 after fitting were excluded from this analysis because there is very little tissue signal for which the diffusion parameters correspond to, which leads to unstable estimations of the parameters.

## Results

### Synthetic dMRI simulations

**FIGURE 2:**
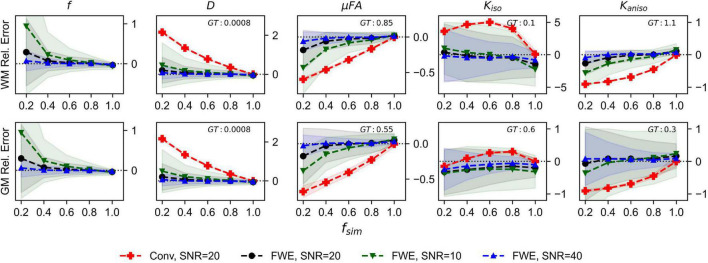
Relative error in diffusion MRI indices measured in synthetic white matter (WM) and gray matter (GM) voxels at various SNR levels and a free water diffusivity of 3*e*−3*mm*^2^/*s*. The *x*-axis depicts the simulated volume fraction (*f*_*sim*_), while the ground truth value for each metric is denoted as GT. The red line with crosses indicates the mean measurements made using the conventional (Conv) method at SNR = 20, the black line with circles depicts the FWE method at SNR = 20, the green line with inverted triangles depicts the FWE method at SNR = 10, and the blue line with triangles depicts the FWE method at SNR = 40.

The relative errors of measurements made with the FWE and conventional techniques at different SNR levels are depicted in Figure 2. Note that the for the conventional method, only the 20 SNR case is displayed because relative errors did not differ by more than the plot line thickness at the various SNR levels. For all volume fractions except *f*_*sim*_ = 1, and at all three SNR levels, the FWE μFA method yielded more accurate mean measurements of *D_T_* and μFA than the conventional method in both the WM and GM configurations. At *f*_*sim*_ = 0.2, the FWE method substantially overestimated *f* in both the WM and GM simulations; however, resulting *D_T_* and μFA estimates were closer to the ground truth than measurements produced by the conventional method. FWE estimates of *K*_*aniso*_ were higher than estimates produced by the conventional method across all *f*_*sim*_, while estimates of *K*_*iso*_ were lower. The variance of parameter estimations over the 1,000 repetitions increased for decreasing *f*_*sim*_.

**FIGURE 3:**
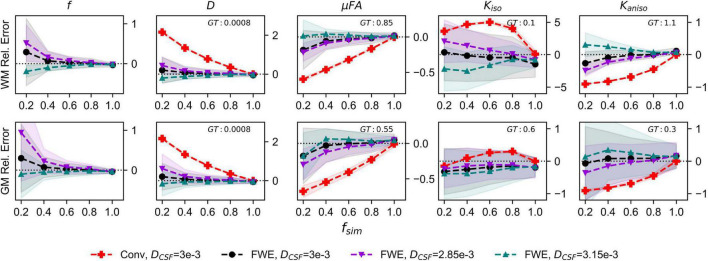
Relative error in diffusion MRI indices measured in synthetic white matter (WM) and gray matter (GM) voxels with various free water diffusivities (*D*_*CSF*_) and an SNR of 20. The *x*-axis depicts the simulated volume fraction (*f*_*sim*_), while the ground truth value for each metric is denoted as GT. The red line with crosses indicates the mean measurements made using the conventional (Conv) method with *D*_*CSF*_ = 3*e*−3*mm*^2^/*s*, the black line with circles depicts the FWE method with *D*_*CSF*_ = 3*e*−3*mm*^2^/*s*, the purple line with inverted triangles depicts the FWE method with *D*_*CSF*_ = 2.85*e*−3*mm*^2^/*s*, and the teal line with triangles depicts the FWE method with *D*_*CSF*_ = 3.15*e*−3*mm*^2^/*s*.

The relative errors of measurements made with the FWE and conventional techniques using different free water diffusivities are depicted in Figure 3. Note that for the conventional method, only the 3*e*−3*mm*^2^/*s* case is displayed because relative errors did not differ considerably regardless of free water diffusivity. The FWE μFA method again yielded more accurate mean measurements of *D_T_* and μFA than the conventional method in both WM and GM configurations for all free water diffusivity values and across all volume fractions except *f*_*sim*_ = 1. *f* was again overestimated at *f*_*sim*_ = 0.2, with the greatest relative error being observed in the signal with a simulated free water diffusivity of 2.85*e*−3*mm*^2^/*s*. FWE estimates of *K*_*aniso*_ were again higher than estimates produced by the conventional method across all *f*_*sim*_, while estimates of *K*_*iso*_ were lower.

### Monte Carlo simulations

**FIGURE 4:**
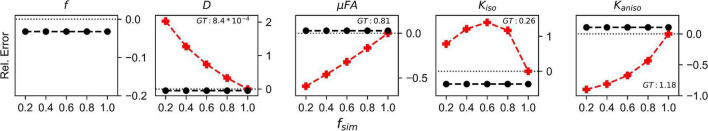
Relative error in diffusion MRI indices measured using the conventional (Conv) and FWE methods on signals simulated using a Monte Carlo technique. The geometry consisted of infinitely long cylinders with a 1 μm radius and 0.7 intra-tube fraction. The *x*-axis depicts the simulated volume fraction (*f*_*sim*_), while the ground truth value for each metric is denoted as GT. The red line with crosses indicates the measurements made using the conventional (Conv) method while the black line with circles indicates measurements made using the FWE method.

The relative errors of measurements made with the FWE and conventional techniques in the Monte Carlo simulations are depicted in Figure 4. Across all *f*_*sim*_, the FWE method underestimated *f* by approximately 3%. At *f*_*sim*_ = 1, measurements of *D_T_* and *K*_*iso*_ made using the FWE method were underestimated by approximately 4.8 and 35%, respectively, relative to measurements made using the conventional method, while measurements of μFA and *K*_*aniso*_ were overestimated by 2.8 and 10.5%, respectively. Measurements made with the FWE technique were consistent across all *f*_*sim*_, while the relative error in all measurements made with the conventional technique increased with decreasing *f*_*sim*_ (except *K*_*iso*_ error, which appeared to peak at a volume fraction in the range of 0.4 < *f*_*sim*_ < 0.6). All metrics measured with the FWE method were much closer to the ground truth than those measured with the conventional method at *f*_*sim*_ < 1.

### In vivo

**FIGURE 5:**
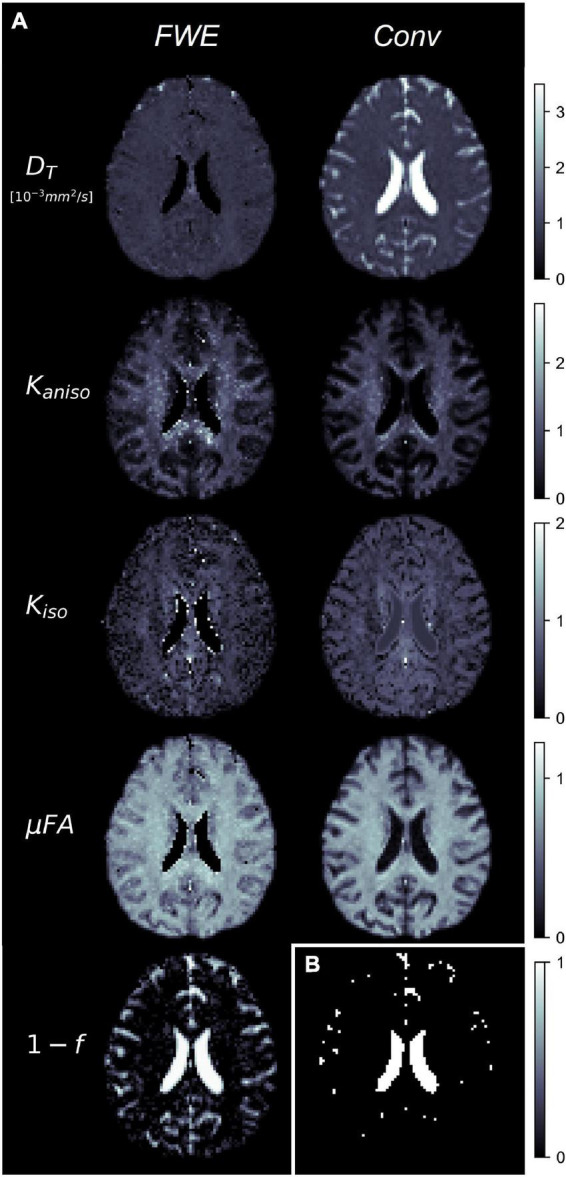
**(A)** Example slices of tissue diffusivity (*D_T_*), anisotropic kurtosis (*K*_*aniso*_), isotropic kurtosis (*K*_*iso*_), microscopic fractional anisotropy (μFA), and fluid volume fraction (1-*f*) measured in one of the healthy volunteers. The images on the left were computed using the free water elimination (FWE) method while those on the right were computed using the conventional (Conv) method. Note that *D_T_* is used interchangeably with *D* for the conventional method. **(B)** Sample slice depicting a binary map showing voxels with tissue volume fractions less than 0.25, which were omitted in region-of-interest analyses.

**FIGURE 6:**
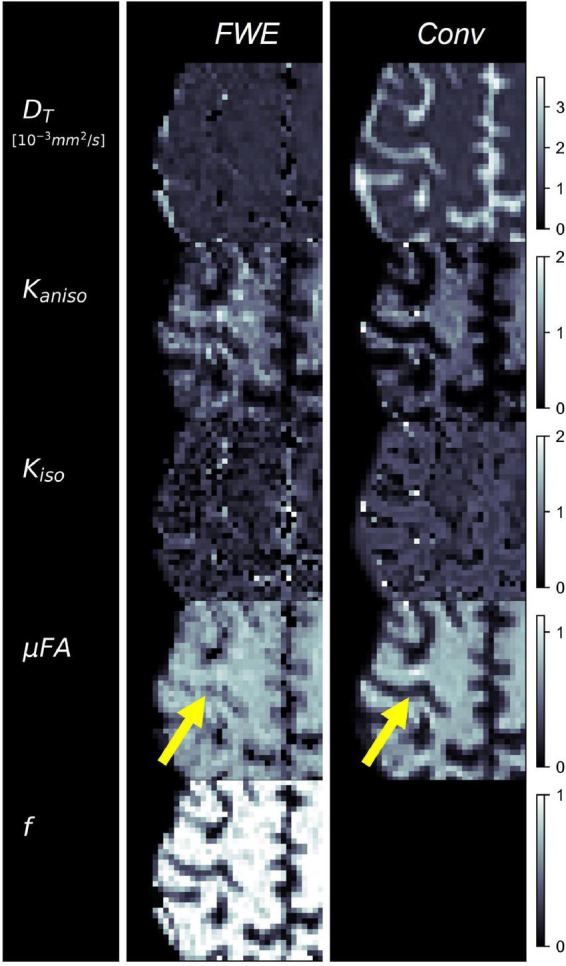
Example cerebral cortex images of tissue diffusivity (*D_T_*), anisotropic kurtosis (*K*_*aniso*_), isotropic kurtosis (*K*_*iso*_), microscopic fractional anisotropy (μFA), and tissue volume fraction (*f*) measured in one of the healthy volunteers. The images on the left were computed using the free water elimination (FWE) method while those on the right were computed using the conventional (Conv) method. Note that *D_T_* is used interchangeably with *D* for the conventional method. The yellow arrow highlights a region in which a significant difference is observed between the FWE and conventional μFA measurements due to high free water contamination.

**FIGURE 7:**
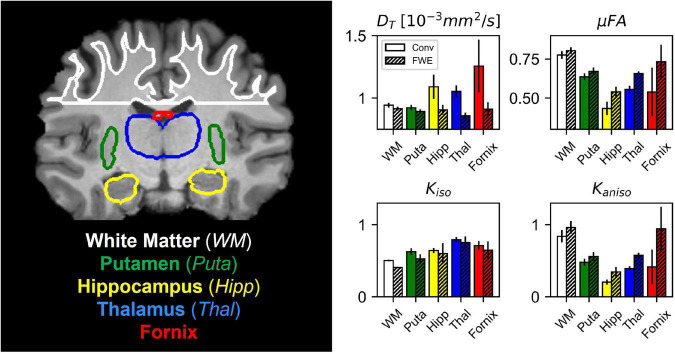
Comparison between the conventional (Conv) and free water elimination (FWE) methods in four healthy volunteers. Depicted on the left is a coronal T1-weighted MPRAGE slice from one of the volunteers highlighting the five regions-of-interest (ROIs). Note that volumetric ROIs were used, despite the single slice depiction. On the right are plots comparing the mean diffusivity (*D_T_*), microscopic fractional anisotropy (μFA), isotropic kurtosis (*K*_*iso*_), and anisotropic kurtosis (*K*_*aniso*_) produced by the Conv and FWE methods. In all ROIs, *D_T_* and *K*_*iso*_ were reduced when FWE was applied, while μFA and *K*_*aniso*_ were elevated, though the magnitude of this difference varied by region. Note that *D_T_* is used interchangeably with *D* for the conventional method.

Example slices of *D_T_*, *K*_*aniso*_, *K*_*iso*_, and μFA generated with the FWE and conventional methods are depicted in Figure 5, as well as a sample slice depicting voxels with *f* < 0.25. Zoom-ins of a cortical region are depicted in Figure 6, where decreases in *K*_*iso*_ and *D_T_*, and increases in μFA and *K*_*aniso*_, are observed for FWE relative to the conventional method throughout the cortex, which agrees with expectations from the simulations. The ROIs are depicted in T1-weighted images in Figure 7, as well as the mean and standard deviations of relevant diffusion indices generated using the two methods. Mean volume fractions in the WM, putamen, hippocampus, thalamus, and fornix regions were 0.96, 0.96, 0.82, 0.82, and 0.64, respectively. Differences in *D_T_* and μFA between the two methods were smallest in the WM and putamen ROIs. When the FWE method was employed, *D_T_* was reduced by 6.4 and 7.5% in the WM and putamen, respectively, compared to measures made using the conventional method, while μFA was increased by 3.5 and 5.3%. Greater differences between methods were observed in the deep GM regions: *D_T_* was reduced by 37.1% in the hippocampus and 42.8% in the thalamus when FWE was used, while μFA was increased by 22.0 and 16.8% in those regions. The most significant differences between methods were observed in the fornix, in which *D_T_* was reduced by 59.2% and μFA was increased by 30.5% when FWE was applied. In all ROIs, mean *K*_*aniso*_ was reduced while mean *K*_*iso*_ was increased when FWE was used.

## Discussion

The FWE method presented herein allows for rapid computation of free water-corrected μFA because it uses alternating least squares estimations for *f* and the various diffusion parameters, which are individually rapid. The total processing time was < 1 min for each subject on a common personal desktop computer. In this work, 100 iterations were performed for each step, but computation time could be further reduced by setting termination criteria for instances in which 100 iterations would be excessive. One such example would be to use the estimate of *f* from Part 1 to omit voxels with very high CSF contamination (e.g., *f* < 0.25) from Part 2.

In synthetic dMRI simulations, the FWE method produced more accurate measurements of *D_T_* and μFA than the conventional method across all volume fractions except *f*_*sim*_ = 1. At *f*_*sim*_ = 1, the simulated signal vs. b-value curve has no free water component and resembles the paK signal representation (equations 5 and 6), so the two-compartment representation is redundant and falsely detects a small free water compartment due to the added noise. In simulations with no added noise (data not shown), the FWE and conventional methods both correctly measure *D_T_* and μFA at *f*_*sim*_ = 1, though only the FWE method yields correct indices at lower *f*_*sim*_.

The increase in *K*_*aniso*_ when the FWE method was employed can be explained by the fact that *K*_*aniso*_ arises solely from the tissue compartment. *K*_*aniso*_ describes the variance in the dMRI powder average signal due to the eccentric shape of neuron fibers and other compartments (Jones et al., 2013); for example, a dMRI acquisition in the direction parallel to neuronal axons will yield a lower signal than one perpendicular to the axons. By removing the isotropic free water compartment, the effect of *K*_*aniso*_ on the remaining signal component is amplified. The reduction in *K*_*iso*_ when the FWE method was used can be attributed to the fact that *K*_*iso*_ describes the variance in diffusivity across compartments; thus, removing the free water compartment, which contains a significantly higher mean diffusivity than neural tissue, attenuates this metric.

Comparisons between measurements made at different SNR values revealed that the FWE technique is susceptible to noise, as mean measurements accuracy decreased and standard deviation across 1,000 voxels increased with decreasing SNR. Despite its sensitivity to noise, the FWE technique still produced more accurate mean measurements at the low SNR of 10 than the conventional method did at any SNR level in regions with reduced *f*_*sim*_. These results suggest that the effects of free water partial volume contamination can be more deleterious to dMRI measurements than noise at the SNR levels typically achieved *in vivo*.

Synthetic dMRI simulations assessing the effects of deviations in the assumed free water diffusivity revealed that measurements made with the FWE technique are generally less accurate when the diffusivity of free water is not exactly 3*e*−3*mm*^2^/*s*. In real tissue, deviations from the assumed temperature of 37°C and biases due to differences in T1 and T2 can alter the free water diffusivity and affect the accuracy of the signal fitting algorithm (Pasternak et al., 2009, 2014b). However, this limitation is shared by all multi-compartment signal representations that use fixed estimates of free water diffusivity and can only be overcome by determining the value prior to the fitting or by attempting to solve for the free water diffusivity in each voxel as an additional variable at the expense of computation time and potential misestimation. Note that despite this limitation, the FWE method still yielded more accurate mean measurements than the conventional method at lower *f*_*sim*_.

In the Monte Carlo simulations, the FWE method underestimated *f* by a relatively constant 3% for all simulated tissue volume fractions. At *f*_*sim*_ = 1, the water-containing cylinders comprise of 70% of the simulated volume, but the extra-tube water is restricted by their presence and likely contributes to *D_T_*. This bias likely resulted from kurtosis arising from the simulation geometry being partially misattributed to a free water compartment. Nevertheless, the bias is small and consistent for different volume fractions, which mitigates deleterious effects when comparing different regions or subjects. Note that repeating the Monte Carlo simulation with 60 directions at each b-value instead of 15 did not reduce the bias in *f*, which suggests that an inadequate number of directions in the powder average was not the cause.

The FWE method also showed promising results when used to measure *D_T_* and μFA in healthy volunteers as differences between the two methods in the various ROIs agreed with expectations. In all ROIs, *D_T_* was reduced and μFA was elevated when the FWE method was used (Figure 7); these changes are intuitive as removing an isotropic signal compartment with high diffusivity from the overall signal, which also contains anisotropic signal components from neurites and other eccentric compartments, will raise the measured diffusion anisotropy and lower the mean diffusivity. The results of the *in vivo* imaging analysis agreed with the hypotheses that the effects would be smallest in the WM and putamen regions and greatest in the free water-adjacent fornix ROI (Figure 7). Furthermore, the *f* parameter allowed for the removal of voxels with high CSF contamination from the ROI analysis, improving mean measurements. However, one drawback of the technique is that there are no ground truth measurements to validate the measured indices against. Comparing measured tissue volume fractions against known values from the literature can act as a pseudo-validation of the FWE method, though it should be noted that the measured *f* index represents the T2-weighted signal fraction of the tissue compartment rather than the true volume fraction. To convert *f* to the true volume fraction of tissue, *f_T_*, a correction can be made as per the following equation (Veraart et al., 2018):


(14) $$f=\frac{f_{T}\,e^{-T\,E/T\,2_{T\,i\,s\,s\,u\,e}}}{f_{T}\,e^{-T\,E/T\,2_{T\,i\,s\,s\,u\,e}}+\left(1-f_{T}\right)\,e^{-T\,E/T\,2_{C\,S\,F}}}$$

Literature reports free water volume fractions of < 2% for WM and 7–9% for GM with high standard deviations (Ernst et al., 1993; Bender and Klose, 2009), which correspond to *f_T_* values of > 0.98 for WM and 0.91–0.93 for GM. Assuming *T2*_*CSF*_ = 1,250 ms (Piechnik et al., 2009), *T2*_*WM*_ = 70 ms (Stanisz et al., 2005), and *T2*_*Putamen*_ = *T2*_*Hippocampus*_ = *T2*_*Thalamus*_ = 95 ms (Stanisz et al., 2005; Bartlett et al., 2007) at 3T, the approximate mean *f_T_* values for the WM, putamen, hippocampus, and thalamus regions were 0.99, 0.98, 0.92, and 0.92 in the healthy volunteers imaged in this work. As expected, the volume fraction in the fornix was measured to be much lower than the other ROIs (*f* = 0.64); no correction was performed for this region because many voxels contained large volumes of pure CSF, which violates the assumptions of equation 14. While previous studies have found evidence that brain tissue volume fraction decreases with age due to increased interstitial space (Chad et al., 2018), such effects are not expected to have impacted the results of this work due to the young age of the participant cohort.

There are several limitations potentially affecting this study. Diffusion time discrepancies between the LTE and STE sequences, and between the three gradient channels in the STE sequence, were not taken into consideration in this work. Different diffusion times in the LTE and STE acquisitions could lead to slight differences between the respective powder average signals that are misattributed to be differences between *K*_*LTE*_ and *K*_*STE*_, while different diffusion times in the different gradient channels for the STE acquisitions could give rise to orientational biases (Jespersen et al., 2019). These potential biases are not expected to have had a significant effect on the results of this work since both the FWE method and conventional method were applied to the same data, and any biases caused by time-dependent diffusion would affect both approaches. However, future studies should consider using optimized STE sequences to ensure that the diffusion time of the STE and LTE sequences match and that there are no orientational biases in the STE sequence.

Another limitation is that the time required for an acquisition protocol to acquire powder average signals at 4 b-values in both LTE and STE schema could be prohibitively long for some applications (our *in vivo* scan required 9 min).

Both the conventional and FWE approaches used herein assume that any deviation from mono-Gaussian diffusion in the tissue arises exclusively from two distinct sources: *K*_*aniso*_ and *K*_*iso*_. However, restricted diffusion inside compartments, exchange between compartments, and microstructural disorder can also contribute to the overall kurtosis and are often categorized together in a term known as microscopic kurtosis (*K*_μ_) (Jespersen et al., 2019; Henriques et al., 2020). Though most μFA imaging techniques do not consider *K*_μ_, recent studies have found that it is non-negligible in the human brain and that ignoring it can lead to biases (Novello et al., 2022). Despite this limitation, μFA techniques that do not distinguish *K*_μ_ from other kurtosis sources have shown promising diagnostic and research capabilities and still represent a significant advance over the widely used DTI metrics.

The images produced by the FWE method (Figures 5, 6) appear grainier than those produced by the conventional method and higher standard deviations were measured in all metrics when the FWE method was used, both in simulations (Figures 2, 3) and *in vivo* (Figure 7). This increased variance is expected due to the increased complexity of the FWE-pAK representation relative to the paK representation. Studies that use the FWE technique should design MRI protocols that sample more b-shells to improve the data fit and acquire more LTE and STE scans at each b-value to raise the SNR of the powder average signals. A minimalistic protocol, such as those described in the literature (Nilsson et al., 2019; Arezza et al., 2021), may be insufficient for FWE imaging. Also, regularization enforcing spatial smoothness, similar to that applied in other applications of FWE, could likely help mitigate this issue (Pasternak et al., 2009; Golub et al., 2020).

The conventional and FWE methods used in this work both derive μFA and other metrics using orientationally-averaged signals in the LTE scheme, which can introduce biases into measurements due to the positively-skewed distribution of Rician noise (Gudbjartsson and Patz, 1995; Aja-Fernandez and Vegas-Sanchez-Ferrero, 2018) and due to non-uniform or insufficient sampling of the diffusion sphere in the LTE regime (Afzali et al., 2021). While measures were taken to denoise and preprocess the *in vivo* dMRI data used in this work, some of the LTE b-shells were minimalistic (e.g., only six directions were acquired at 1,400 s/mm^2^). The simple arithmetic averaging method used in this work to compute powder average signals may be suboptimal given the minimalistic LTE acquisition protocol used, and more advanced algorithms to compute the powder average signal could potentially reduce biases (Afzali et al., 2021).

In conclusion, the two-compartment μFA imaging technique presented in this work represents an extension to a conventional μFA imaging technique that integrates a free water compartment to extract tissue-specific indices of *D*, *K*_*aniso*_, *K*_*iso*_, and μFA. This approach requires only modest assumptions about the content of the voxel and makes no assumptions about the tissue microstructure–it could be described as a “macrostructural model.” To solve the ill-conditioned fit of the data to equations (8) and (9), a two-part algorithm was employed to first determine initial guesses of key parameters and then to perform the joint fit. Any dMRI protocol designed to estimate μFA *via* the FWE method proposed in this work will be versatile due to the need for multiple b-shells in both LTE and STE schema and can also be fitted to the conventional μFA method and the DKI signal representation; furthermore, if a significant number of LTE directions are acquired at *b* = 1,000 s/mm^2^, the data can be fitted to the widely adopted DTI signal representation as well. Both simulation and real data experiments indicated that the FWE method may be a feasible technique for measuring μFA and other dMRI indices with greater specificity to neural tissue characteristics by removing free water partial volume effects. It should also be noted that other μFA approaches, such as the STE techniques that use the gamma signal model, double diffusion encoding (DDE) techniques, and techniques that exclusively derive the metrics from LTE acquisitions, could likely also be modified to include a free water compartment.

## Data availability statement

Publicly available datasets were analyzed in this study. This data can be found here: https://osf.io/etkgx/.

## Ethics statement

The studies involving human participants were reviewed and approved by the Western University Institutional Review Board. The patients/participants provided their written informed consent to participate in this study.

## Author contributions

NA: conceptualization, methodology, experiments, and writing. TS: methodology and experiments. MO: preliminary experiments. CB: conceptualization, methodology, review, and supervision. All authors contributed to the article and approved the submitted version.

## Footnotes

1
https://github.com/ANTsX/ANTs
